# Sialic acid *O*-acetylation: From biosynthesis to roles in health and disease

**DOI:** 10.1016/j.jbc.2021.100906

**Published:** 2021-06-19

**Authors:** Eline A. Visser, Sam J. Moons, Suzanne B.P.E. Timmermans, Heleen de Jong, Thomas J. Boltje, Christian Büll

**Affiliations:** 1 Institute for Molecules and Materials, Department of Synthetic Organic Chemistry, Radboud University Nijmegen, Nijmegen, the Netherlands; 2 Copenhagen Center for Glycomics, Departments of Cellular and Molecular Medicine, Faculty of Health Sciences, University of Copenhagen, Copenhagen, Denmark; 3 Hubrecht Institute, Utrecht, the Netherlands

**Keywords:** sialic acids, *O*-acetylation, sialic acid O-acetyl transferases (SOATs), sialic acid O-acetyl esterases (SIAEs), influenza C/D virus, glycan modifications, glycobiology, Ac-CoA, acetyl-coenzyme A, ALL, acute lymphoblastic leukemia, BCoV, bovine coronavirus, CASD1, capsule structure1 domain containing 1, CMAH, CMP-Neu5Ac hydroxylase, CMP, cytidine 5′-monophosphate, CPS, capsular polysaccharides, EHEC, enterohemorrhagic *Escherichia coli*, GBP, glycan-binding protein, HA, hemagglutinin, HE, hemagglutinin esterase protein, HEF, hemagglutinin esterase fusion protein, IA/B/C/DV, Influenza A/B/C/D virus, KDN, 2-keto-3-deoxynononic acid, MERS-CoV, Middle East respiratory syndrome coronavirus, MHV-S, murine hepatitis virus strain S, MUC, mucin, NA, neuraminidase, Neu5Ac, N-acetylneuraminic acid, Neu5Gc, N-glycolylneuraminic acid, NPL, neuraminic acid pyruvate-lyase, O-Ac, O-acetyl, S protein, spike protein, SARS-CoV, severe acute respiratory syndrome coronavirus, SDAV, sialodacryoadenitis virus, Sia, sialic acid, SIAE, sialic acid O-acetyl esterase, Siglec, sialic acid-binding immunoglobulin-like lectin, sLe^x^, sialyl-Lewis^x^ antigen, SOAT, sialic acid O-acetyl transferase

## Abstract

Sialic acids are nine-carbon sugars that frequently cap glycans at the cell surface in cells of vertebrates as well as cells of certain types of invertebrates and bacteria. The nine-carbon backbone of sialic acids can undergo extensive enzymatic modification in nature and *O*-acetylation at the C-4/7/8/9 position in particular is widely observed. In recent years, the detection and analysis of *O*-acetylated sialic acids have advanced, and sialic acid-specific *O*-acetyltransferases (SOATs) and *O*-acetylesterases (SIAEs) that add and remove *O*-acetyl groups, respectively, have been identified and characterized in mammalian cells, invertebrates, bacteria, and viruses. These advances now allow us to draw a more complete picture of the biosynthetic pathway of the diverse *O*-acetylated sialic acids to drive the generation of genetically and biochemically engineered model cell lines and organisms with altered expression of *O*-acetylated sialic acids for dissection of their roles in glycoprotein stability, development, and immune recognition, as well as discovery of novel functions. Furthermore, a growing number of studies associate sialic acid *O*-acetylation with cancer, autoimmunity, and infection, providing rationale for the development of selective probes and inhibitors of SOATs and SIAEs. Here, we discuss the current insights into the biosynthesis and biological functions of *O*-acetylated sialic acids and review the evidence linking this modification to disease. Furthermore, we discuss emerging strategies for the design, synthesis, and potential application of unnatural *O*-acetylated sialic acids and inhibitors of SOATs and SIAEs that may enable therapeutic targeting of this versatile sialic acid modification.

Sugars serve as essential molecular building blocks that can assemble into complex glycans with numerous biological functions (1). Virtually every cell produces glycans; short, long, linear, and branched structures composed of different types of sugar molecules that are attached to membrane and secreted glycoproteins and glycolipids. The vast and diverse collection of glycans produced by a cell or tissue is referred to as “the glycome” (2). In vertebrate cells, glycans are assembled inside the endoplasmic reticulum, Golgi system, nucleus, cytoplasm, and mitochondria where over 200 glycosyltransferase enzymes build the glycome (2, 3). The glycome regulates a multitude of biological processes, such as the functional and biochemical properties of proteins and lipids, and cellular adhesion, communication, and immune recognition events (4). Important determinants of the biological functions of glycans are the sialic acids (Sias) that reside at the terminal position of glycans in vertebrate cells, some invertebrates, and some human pathogens. The sialic acid family comprises >80 naturally occurring members that are related to the nonulosonic acids, nine-carbon backbone α-keto sugars that are widely found in nature (5, 6, 7). Regarding this large diversity, the assembly of sialic acid-carrying glycans (sialoglycans) forms a subclass within the glycome—the sialome (8). The diverse biological functions of sialic acids include protection of the cell surface and glycoproteins from proteases (9, 10); regulation of serum half-life of glycoproteins and erythrocytes that are cleared in the liver upon desialylation (11, 12, 13); and likely formation of the blood vessel lumen (14). In the immune system, sialoglycans such as sialyl Lewis^x^ contribute to immune cell trafficking *via* binding to selectins on the endothelium (15), and they form the ligands for the immunomodulatory Siglec receptors that set the threshold for immune activation (16, 17). Sialic acids also serve as binding sites for human pathogens and can be utilized by microorganisms for molecular mimicry (18, 19). Furthermore, aberrant sialoglycan expression is associated with tumor growth, immune evasion, and metastasis (20, 21, 22).

The biological versatility of sialic acids is reflected in their large structural diversity that arises from the natural modifications (Fig. 1*A*) and the different linkages (α2-3/6/8) to underlying glycans and glycoconjugates (N-/O-glycans, glycolipids). The most prevalent sialic acid derivative in humans is N-acetylneuraminic acid (Neu5Ac), and other notable sialic acid derivatives are 2-keto-3-deoxynononic acid (KDN) and N-glycolylneuraminic acid (Neu5Gc) (5). Interestingly, humans have lost the ability to biosynthesize Neu5Gc due to a mutation in the CMP-Neu5Ac hydroxylase (*CMAH*) gene (23); however, Neu5Gc can be scavenged from exogenous, dietary sources, and low amounts of this derivative are incorporated into the sialoglycans of human cells (24). Further modifications at any of the hydroxyl or amine groups of the sialic acid backbone result in the >80 distinct naturally occurring sialic acid types known to date (6, 7). Analysis of these sialic acid types in biological samples is challenging, and often their biosynthesis and biological functions are not fully understood. Presumably, the extensive Sia modifications may be the result of an evolutionary race between the host and pathogens that exploit sialoglycans for infection. In line with the Red Queen hypothesis, an evolutionary biology concept in which the host species must constantly adapt to survive competition with evolving pathogens, Sia modifications may have evolved to abrogate pathogen interactions while preserving overall endogenous functions in the host (25). Moreover, the modifications may contribute additional regulatory or informational cues to sialoglycan recognition that are advantageous to the host.

**Figure 1:**
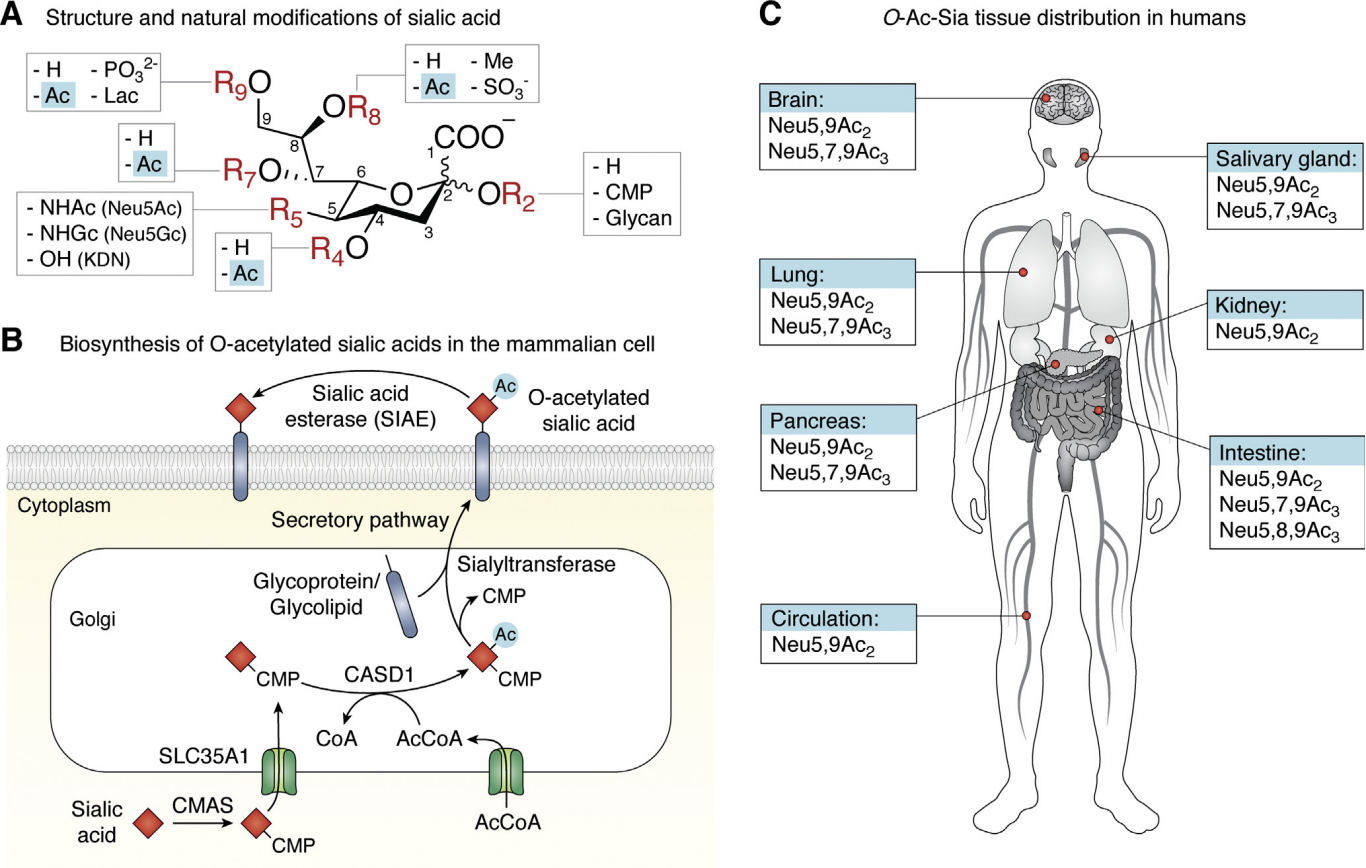
**Structure, biosynthesis, and distribution in humans of *O*-acetylated sialic acids.***A*, structure of the nine-carbon (C-1 to C-9) sialic acid backbone and the natural modification sites (R2, R4-R9) with naturally occurring modifications indicated. Modifications at C-5 form the frequent sialic acid types N-acetylneuraminic acid (Neu5Ac), 2-keto-3-deoxynononic acid (KDN), and N-glycolylneuraminic acid (Neu5Gc). *O*-acetylation (Ac) can occur at the positions C-4, and C-7/8/9. *B*, presentation of the biosynthetic pathway of *O*-Ac-Sias in mammalian cells. Sialic acids (*red diamond*) are conjugated to cytidine 5′-monophosphate (CMP) *via* the nuclear enzyme CMAS and are transported into the Golgi system *via* the CMP-sialic acid transporter SLC35A1. CASD1 acetylates CMP-sialic acids at position C-7 or C-9 using Ac-CoA as acetyl donor. Sialyltransferases conjugate *O*-acetylated sialic acids (with CMP as leaving group) to the glycans of glycoproteins and glycolipids that exit the Golgi system *via* the secretory pathway. For so far unknown reasons, glycoproteins and glycolipids carrying *O*-acetylated sialic acids appear also to remain inside the Golgi system. Deacetylation is mediated by the sialic acid esterase SIAE that is localized extracellularly and in intracellular compartments. *C*, tissue distribution of *O*-acetylated sialic acids in the human body.

A common natural sialic acid modification is the addition of one or more *O*-acetyl esters to the hydroxyl groups of sialic acid residues yielding about 20 different naturally occurring *O*-Ac-Sias (26). Due to advances in the detection and analysis of *O*-Ac-Sias and the identification of SOATs and SIAEs in mammalian cells, invertebrates, bacteria, and viruses, many aspects regarding the biosynthesis and roles in biology and disease of this modification have recently been uncovered, and we anticipate that this rapid pace of discovery will continue. Here, we review the current understanding of the biosynthesis of *O*-acetylated sialic acids and their roles in health and disease and discuss implications for the design, synthesis, and emerging applications of sialic acid *O*-acetylation inhibitors.

## Biosynthesis and tissue distribution of *O*-Ac-Sias

### *O*-acetylated sialic acids

Soon after the discovery of sialic acids by Blix and Klenk (27, 28), the presence of *O*-acetyl modifications was noted, and among others, Schauer and Kamerling succeeded in their verification using mass spectrometry (6, 29). *O*-acetylation can occur at the C-4, C-7, C-8, and C-9 hydroxyl groups of the nonulosonic acid and sialic acid backbone (Fig. 1*A*). These modifications are denoted as exemplified for Neu5Ac (which carries an N-acetyl group at C-5) as Neu4,5Ac_2_, Neu5,7Ac_2_, Neu5,8Ac_2,_ and Neu5,9Ac_2,_ respectively (30). *O*-acetylation can also occur simultaneously at multiple positions, giving rise to di- and tri-*O*-acetylated Sias such as Neu5,7,9Ac_3_ or Neu5,7,8,9Ac_4_, respectively. An overview of the identified naturally occurring *O*-Ac-Sias is provided by Varki, Schauer, and Kamerling, who made seminal contributions to their identification and biology (6, 7, 31). *O*-acetylation is not static, as spontaneous migration of the *O*-acetyl groups over the glycerol chain can occur, but not to or from the C-4 position (32). At neutral and slightly basic pH, bidirectional *O*-acetyl group migration along the glycerol tail from C-7, C-8, and C-9 was observed yielding Neu5,7Ac_2_, Neu5,8Ac_2_, and Neu5,9Ac_2_, respectively (32, 33). In addition, the formation of di-*O*-acetyl-Sia Neu5,8,9Ac_3_ from Neu5,7,9Ac_3_ has been indicated (32, 34). Sia *O*-acetyl group migration can be bidirectional and proceeds intramolecularly through orthoester intermediates, which mainly takes place at neutral and mild basic pH while it stabilizes at mildly acidic pH <5 (32, 33). Recent advances in sample preparation and analysis of *O*-Ac-Sia species now provide opportunity to address the migration process of the acetyl group (35, 36, 37).

### Biosynthesis

The biosynthesis of *O*-Ac-Sias in mammalian cells starts with sialic acids that are produced *de novo* in the cytoplasm *via* several enzymatic steps or derived from exogenous sources (*e.g.*, dietary) (38, 39). Sialic acids are activated in the nucleus by conjugation to cytidine 5′-monophosphate (CMP) and transported into the Golgi system where 20 sialyltransferase isoenzymes use CMP-sialic acids as donor to incorporate sialic acids into glycans *via* distinct glycosidic linkages (α2-3/6/8) (2, 40). *O*-acetylation takes place in the Golgi system and involves the activity of sialic acid *O-*acetyltransferases (SOATs) and sialic acid esterases (SIAEs) that add and remove *O*-acetyl groups on sialic acids, respectively (26) (Fig. 1*B*). So far, a single mammalian SOAT (CASD1, capsule structure1 domain containing 1) has been identified (41, 42), and one sialic-acid-specific esterase (SIAE) is known (43, 44, 45, 46, 47) (Table 1). Especially the identification of CASD1 remained challenging for several decades due to lability of the intact enzyme during purification (48, 49, 50, 51, 52). Eventually, mammalian SIAE was cloned in 1996 (43), whereas CASD1 was identified 2011 through data mining of the human genome for acetyltransferase genes with unknown functions (41) and biochemically characterized in 2015 (42).

**Table 1:** Occurrence of *O*-Ac-Sias and biosynthetic enzymes in humans and microorganisms

Species	*O*-Ac-Sia form	SOAT	SIAE	*O*-Ac-Sia GBP	References
*Homo sapiens*	Diverse (Fig. 1*C*)	CASD1	SIAE	nd	(41, 42, 43, 44)
*Campylobacter jejuni*	Neu5,9Ac_2_ (α2-8-linked)^a^	Orf11	nd	nd	(190)
*Escherichia coli* K1	Neu5,7/9Ac_2_ (α2-3/8-linked)^a^	NeuO NeuD	NeuA	nd	(194, 195, 196, 197, 198)
Enterohemorrhagic *E. coli* (EHEC)	Neu5,9Ac_2_	NA	NanS NanS-p	nd	(167, 168, 169, 170, 171)
*Neisseria meningitides,* serogroup C	Neu5,7/8Ac_2_ (α2-9-linked)^a^	OatC	nd	nd	(191, 201)
*Neisseria meningitides,* serogroups W-135 and Y	Neu5,7/9Ac_2_ (α2-6-linked)^a^	OatWY	nd	nd	(192, 201, 202)
*Streptococcus agalactiae* (Group B *Streptococcus*)	Neu5,7/8/9Ac_2_ (α2-3-linked)^a^	NeuD	NeuA	nd	(189, 203, 204)
*Streptococcus pneumoniae*	nd	nd	Axe EstA	nd	(175)
*Tannerella forsythia*	Neu5,9Ac_2_	nd	NanS	nd	(176, 177)
*Salmonella typhi*	Neu5,9Ac_2_	nd	nd	Typhoid toxin	(179, 182)
*Leishmania donovani*	Neu5,9Ac_2_	nd	nd	nd	(210, 211)

Identified *O*-acetylated sialic acids SOATs, SIAEs, and GBP with specificity for *O*-Ac-Sias are presented.

Abbreviations: GBP, glycan-binding proteins; nd, not determined.

a Capsular *O*-Ac-Sia.

Human CASD1 is a multimembrane-spanning protein localized in the Golgi system and has preferred substrate specificity for CMP-sialic acids, thus *O*-acetylation likely occurs prior to the transfer by sialyltransferases (42). However, *O*-acetylation may also occur after sialylation (53), and the ability of CASD1 to *O*-acetylate different sialoglycan types should be investigated. Human CASD1 is a homologue of the *Cryptococcus neoformans* Cas1p protein that *O*-acetylates yeast glycans (54), and further detailed phylogenetic analysis should map CASD1 and its roles in glycan modification throughout evolution. The catalytic domain of CASD1 in the Golgi lumen is predicted to adopt an α/β-fold typical for GDSL/SGNH-like acyl-esterase family members and is formed by catalytic serine (S94), aspartic acid (D270), and histidine (H273) residues (42, 55). Although CASD1 displays a fold common for acyl-esterases, CASD1 was shown not to act as an esterase for 9-*O*-Ac-Sia nor *p*-nitrophenyl acetate (42). Instead, CASD1 was demonstrated to acetylate CMP-sialic acids *via* a “ping-pong” mechanism proceeding through a covalent acetyl-serine intermediate. The acetyl donor is acetyl-coenzyme A (Ac-CoA) that is transported into the Golgi (*via* Acetyl-coenzyme A transporter 1, SLC33A1) where it is utilized by CASD1 for *O*-acetylation of the C-7/9 position of CMP-sialic acid, with ability to migrate along the glycerol chain (42, 56). Also 4-*O*-acetylation capacity has been indicated in mammalian cells (57, 58), but the mechanism and a specific SOAT remain to be identified.

*O*-Ac-Sias are incorporated into the different glycoconjugates by the sialyltransferase isoenzymes. Studies with cell lines and mice suggest that ST6GAL1 installs Neu5,9Ac_2_ on N-glycans (59), ST3GAL1 adds *O*-Ac-Sia to *O*-glycans (60), and ST3GAL5 and ST8SIA1 can transfer *O*-Ac-Sia to gangliosides that can carry *O*-Ac-Sia on the terminal and possibly on the inner sialic acid (42, 61). While CASD1 and *O*-Ac-Sia are ubiquitously expressed in the Golgi system of mammalian cell lines (62), only select cell lines show surface expression of *O*-Ac-Sia. This suggests that the sialyltransferase isoenzymes with cell-type specific expression have different selectivity for *O*-Ac-Sia. This notion is supported by studies showing that surface *O*-Ac-Sia can be induced by overexpression of select sialyltransferases (42, 59, 63, 64, 65). Another explanation may be that *O*-Ac-Sias are added to specific proteins or lipids expressed at the cell surface or in secretion. This phenomenon should be further investigated by probing *O*-Ac-Sia recognition by the sialyltransferases isoenzymes for instance using cells with combinatorial genetic engineering of the sialyltransferases (3, 66) and by isolation and identification of membrane-bound and secreted *O*-acetylated glycoproteins/glycolipids using *O*-Ac-Sia-specific probes (67). Alternatively, deacetylation at the cell surface or during trafficking could explain why only intracellular *O*-Ac-Sia glycans are found in some cell lines.

Deacetylation of 4/9-*O*-Ac-Sia (7-*O*-acetyl groups are resistant) can be mediated by SIAE. Like CASD1, human SIAE displays the topological features of the SGNH-hydrolase family and mutation of either S127, T132, or H377 all led to inactive SIAE variants demonstrating that these residues constitute the catalytic triad (44, 68). The *SIAE* gene was identified in >200 vertebrate species, and phylogenetic analysis has identified orthologs in invertebrates and bacteria with conserved regions including the catalytic residues (44, 69). SIAE was initially suggested to localize to the lysosome and to contribute to the degradation of *O*-acetylated sialoglycans together with lysosomal sialidases (45); however, more recent colocalization studies suggest localization of SIAE in the Golgi, but not the lysosome (44, 69). Moreover, expression studies with human SIAE and zebrafish Siae, respectively, in COS7 cells showed that both esterases are glycosylated and secreted *via* the secretory pathway into the culture medium (44, 69). It is conceivable that SIAE-mediated deacetylation may occur at different intracellular and extracellular locations to serve site-specific functions, for example, in the degradation and recycling of Sias in the lysosome, regulation of sialoglycan *O*-acetylation in the Golgi, or unmasking of cell surface and circulating sialoglycoproteins by the secreted esterase. The cellular localization and (site-)specific functions of SIAE require further characterization to understand the regulation and molecular functions of deacetylation. Beyond SIAE, other nonspecific esterases or other factors (*e.g.*, pH) could lead to the deacetylation of *O*-Ac-Sias. Overall, many aspects regarding the biosynthesis of *O*-Ac-Sias remain poorly understood. Are there other mammalian SOATs and SIAEs except from CASD1 and SIAE and what is the evolution of these enzymes? Is there a C-4-specific SOAT in humans? How are Sia *O*-acetylation and deacetylation as well as migration over the glycerol chain regulated? Why are *O*-Ac-Sias ubiquitously expressed in the Golgi system, but not at the cell surface? Is there selectivity of sialyltransferase isoenzymes for *O*-Ac-Sia and thus glycoconjugate-specific and potentially glycoprotein-specific expression?

### Tissue distribution

*O*-acetylation is presumably the most widespread sialic acid modification in nature, and like sialic acids can be found across vertebrates, echinoderms, protozoa, fungi, and various bacteria (7, 26). *O*-Ac-Sias can be detected with mass spectrometry, liquid chromatography (HPLC), and immunohistochemistry using antibodies or probes derived from viral lectins (virolectins) (33, 36, 37, 58, 67, 70). Further technical advances will overcome challenges in the analysis of *O*-Ac-Sias (isolation, lability, migration, intracellular localization) and enable comprehensive studies into their species, tissue, and cell-type-specific distribution and functions. Generally, *O*-Ac-Sia can be found throughout the human body, with high expression in the human colon, which is in line with CASD1 and SIAE expression levels that are highest in the digestive and gastrointestinal tract, brain, and endocrine tissues (Human Protein Atlas) (71). 9-*O*-Ac-Sias seem to be most frequent, and 4-*O*-Ac-Sias appear the least frequent (6); however, little is known about the latter modification in humans. 4-*O*-Ac-Sias have not been detected in human tissue and human cell lines (HEK293, A549) with virolectin (58), but were found in human erythrocytes (72) and traces were detected in the intestine (73) using mass spectrometry. An overview of the *O*-Ac-Sia tissue distribution is provided in Figure 1*C*.

## Development and cancer

### Development

**Figure 2:**
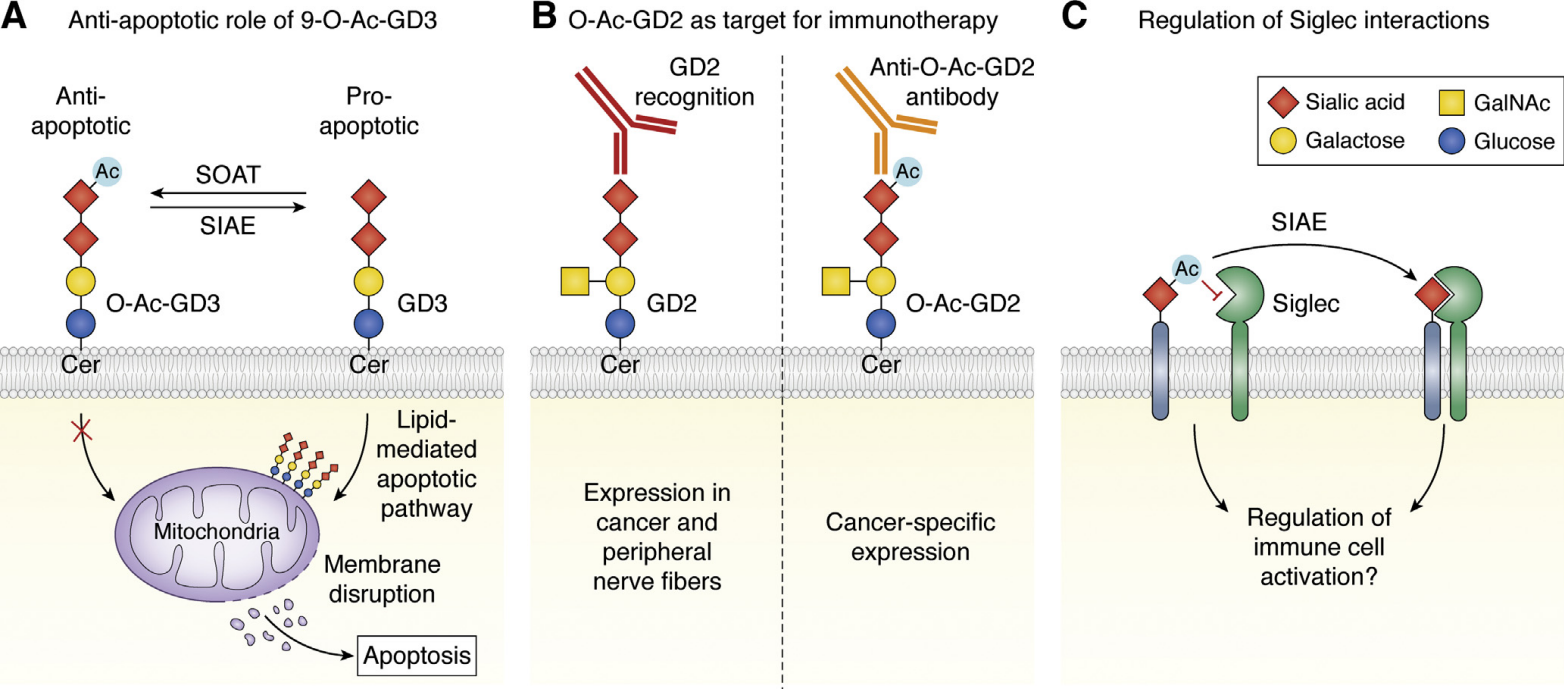
***O*-acetylation of sialic acids as regulator of apoptosis and immune recognition.***A*, the plasma membrane-resident, non-*O*-acetylated form of GD3 can be internalized and accumulates in the mitochondrial membrane. This accumulation results in disruption of the mitochondrial membrane and induces lipid-mediated apoptosis. 9-*O*-acetylation of GD3 prevents internalization and the apoptotic effects of GD3. *B*, the ganglioside GD2 is a clinical target for monoclonal antibody therapy (Dinutuximab, *red*, *left side*) in neuroblastoma, but additional GD2 expression on peripheral nerve fibers causes adverse effects. The *O*-Ac form of GD2 (*O*-Ac-GD2) is more specifically expressed by neuroblastoma cells and targeting this form with specific anti-*O*-Ac-GD2 antibodies (*orange*, *right side*) could circumvent adverse effects. *C*, *O*-acetylation largely inhibits sialic acid binding to immune receptors such as the Siglecs (*red inhibitory arrow*). (De-)*O*-acetylation may serve as on/off-switch in immune signaling. Glycan symbols are drawn according to the symbol nomenclature for glycans (SNFG) (30).

Studies in mice and rats strongly indicate that Neu5,9Ac_2_ carried by GD3, a ganglioside with two Sia residues (Neu5Acα2-8Neu5Ac) linked to lactosylceramide (74), serves important roles in development and differentiation. The 9-*O*-acetylated form of GD3 (9-*O*-Ac-GD3) is best characterized and is expressed during embryonic development in the retina and cells of the developing brain such as migrating neuroblasts, but is absent in the adult brain, suggesting involvement in neural development (75, 76, 77). Moreover, 9-*O*-Ac-GD3 expression is induced during neural regeneration in the adult brain and thus may function in neural repair processes (78). The non-*O*-acetylated form of GD3 has proapoptotic effects and its synthesis is stimulated during CD95/Fas-mediated apoptosis (79, 80). Cell membrane-bound and soluble GD3 can traffic inside the cell and accumulate in the membrane of mitochondria (81). This accumulation leads to disruption of the mitochondrial membrane potential and induces caspase-9 activation and apoptosis (82). In contrast, *O*-acetylation of GD3 prevents internalization and accumulation at the mitochondria and does not induce apoptosis, which was reversible through treatment with a viral esterase (83, 84) (Fig. 2*A*). This suggests that *O*-acetylation regulates the anti-/proapoptotic effects of GD3 and that this modification plays an important role in tissue development and regeneration, which is shaped by apoptosis.

Studying the role of *O*-Ac-Sias in organisms is challenging, because knockout of SOATs will broadly remove sialic acid *O*-acetylation, and remarkably, the recently generated *Casd1* knockout mice are viable and fertile, raising questions concerning the importance of this modification in development (85, 86). More specific deletion of the *O*-Ac-Sia carrier will also only provide limited insight into its role in development. For example, knockout of the GD3 synthase *ST8SIA1* will deplete GD3 (and 9-*O*-Ac-GD3), but can result in higher GM3 levels and alterations in the biosynthesis of other gangliosides (GD2, GD1b, *etc*.) (74), which complicates data interpretation. A more elegant approach used expression of viral SIAE in a tissue-specific manner (87). Injections of plasmids encoding influenza C virus 9-*O*-acetylesterase into fertilized mouse egg cells caused development arrest at the two-cell stage. Tissue-specific expression of influenza C virus 9-*O*-acetylesterase under control of the PMNT promoter (adrenal gland, retina) yielded transgenic mice with loss of *O*-Ac-GD3 and abnormal development of the adrenal gland and retina (87). This is remarkable considering that no such abnormalities have been reported for *Casd1* knockout mice and may hint to the existence of other SOATs (85, 86). Although *CASD1* knockout in cell lines depleted all detectable *O*-Ac-Sias (42, 88), other SOATs may exist with redundant functions to CASD1 or the ability to mediate 4-*O*-acetylation (86), and future genome-wide knockout/in studies may identify these. Furthermore, refined systems that allow tissue-specific and potentially tunable *O*-acetylation by controlling SOAT and SIAE expression will be very useful for future discoveries of the biological functions of *O*-Ac-Sias.

### Cancer

Insight into the role of *O*-Ac-Sia in cancer is largely limited to gangliosides that are frequently found in cancer as *O*-acetylated form (89). The best studied example is 9-*O*-Ac-GD3 that has been detected in various cancers including acute lymphoblastic leukemia (ALL) (90, 91), melanoma (92, 93), neuroblastoma (94), medulloblastoma (95), and breast cancer (96, 97). 9-*O*-Ac-GD3 is normally expressed during embryonic development, but rare in healthy adult tissue. Re-expression of 9-*O*-Ac-GD3 in cancer is presumably the result of altered expression of CASD1, SIAE, and glycosyltransferases (*e.g.*, ST3GAL5, ST8SIA1), which was observed in human medulloblastoma samples (95) and ALL (98, 99). The antiapoptotic effects of 9-*O*-Ac-GD3 could benefit tumor cell survival and proliferation (100) (Fig. 2*A*). Accordingly, several studies demonstrated that induction of human or viral sialic acid esterase reduced GD3 *O*-acetylation and enhanced sensitivity to drug-induced apoptosis in cancer cell lines (83, 95, 101, 102). These studies suggest that strategies to reduce 9-*O*-Ac-GD3 could be employed together with apoptosis-inducing drugs for the treatment of cancer. Conversely, high SIAE expression has been indicated as poor prognostic marker in patients with multiple myeloma (103). Clearly, more studies are needed to understand the effects of CASD1 and SIAE activity in cancer.

The specific expression of *O*-Ac-Sia gangliosides in cancer makes them potential targets for immunotherapy (89). The ganglioside GD2 is highly expressed in neuroblastoma with only low expression in the nervous system and is therefore a prime target in neuroblastoma immunotherapy (104). Dinutuximab, a monoclonal antibody that specifically recognizes GD2 and no other ganglioside, showed efficacy in children with neuroblastoma, but also caused adverse effects including neuropathic pain probably due to GD2 expression on peripheral nerve fibers (105). As neuroblastoma cells express *O*-acetylated GD2 (Neu5,9Ac_2_), specific *O*-Ac-GD2 antibodies that react with neuroblastoma cells, but not peripheral nerve fibers, were produced that could overcome the adverse effects of anti-GD2 antibody therapy (106, 107, 108) (Fig. 2*B*). Also other cancer types have been reported to display *O*-Ac-GD2 including breast cancer (109), melanoma (64, 110), and glioblastoma (111), which could also be targeted with anti-*O*-Ac-GD2 antibody therapy. Little is known about *O*-acetylation of other sialoglycans in cancer. There is evidence that ALL lymphoblasts express α2-6-linked Neu5,9Ac_2_, which is different from the α2-8-linked Neu5,9Ac_2_ in GD3, and that this sialic acid modification promotes survival of lymphoblasts (112, 113, 114, 115).

Alterations in *O*-Ac-Sia expression may also affect tumor immunity and metastasis. Increased *O*-acetylation may come at the expense of losing interactions with the immunosuppressive Siglecs. Binding of GD3 to Siglec 7, which inhibits NK cell cytotoxicity, and interactions with Siglec-15, which promote tumor-associated myeloid cells could be compromised by *O*-acetylation (116, 117, 118, 119). On the other hand, loss of sialoglycan-Siglec-7/9 interactions has been suggested to contribute to tumor-promoting inflammation in colorectal cancer (120, 121). This indicates that *O*-acetylation could have different effects on tumor immunity at different stages of tumor development. Notably, *O*-Ac-Sia expression is downregulated in colorectal cancer, probably as a result of reduced SOAT expression (63,122, 123, 124, 125). Reduced *O*-acetylation in colorectal cancer may favor degradation of the epithelial mucus barrier resulting in tumor-promoting inflammation in response to sensing of the microbiome by the epithelial cells (126). In line with this hypothesis, studies in mice show that a defective mucus barrier promotes tumor formation (127, 128). Moreover, reduced *O*-acetylation in colorectal cancer unmasks sialyl-Lewis^x^ (sLe^x^) epitopes that interact with selectins and may facilitate cancer progression and metastasis (129). Thus, *O*-Ac-Sias are potential targets for cancer therapy and the functional consequences of altered *O*-Ac-Sia expression in the different tumor growth stages and tumor types should be further investigated.

## Immune regulation and autoimmunity

*O*-Ac-Sias are expressed throughout the immune system, but we are just beginning to understand their specific roles in immunity. *O*-acetylation increases the hydrophobic character of sialoglycans and can change the biophysical properties of glycoproteins, glycolipids, and the cell membrane. This can alter binding of glycan-binding proteins (lectins) of the immune system and potentially (glycan) antigen recognition (130, 131, 132). For instance, *O*-acetylation negatively affects sialic acid binding by the complement protein factor H (59) and generally precludes recognition of sialoglycans by the Siglec immunoreceptor family (133, 134). The immunoregulatory effects of *O*-acetylation have best been demonstrated for Siglec-2 (CD22) that negatively regulates B cell receptor signaling. Siglec-2 is expressed by human B cells and binding to its preferred sialoglycan ligand (Neu5Acα2-6Galβ1-4GlcNAc) is blocked by 9-*O*-acetylation (135, 136, 137, 138). *O*-acetylation may therefore function as molecular on/off switch for Siglec-2 binding to sialoglycans and inhibitory signaling in B cells (Fig. 2*C*). In line with this concept, Pillai and coworkers identified polymorphisms that impair activity and/or secretion of SIAE in an autoimmunity patient cohort (68). Reduced SIAE activity could lead to accumulation of *O*-acetylated sialoglycans and reduced ligand recognition by the coinhibitory B cell receptor Siglec-2 (CD22) resulting in disturbed B cell tolerance. This notion was initially supported by studies with *Siae* knockout mice that showed enhanced *O*-acetylation of α2-6-linked Sias on N-glycans and defective B cell tolerance formation (138). However, recently it was discovered that the B cell phenotype in the mice was related to unintended disruption of *Dock2*, but not *Siae* (139), and results from genetic association studies investigating the link between SIAE polymorphisms and autoimmunity are conflicting (140, 141, 142, 143). While it is generally accepted that *O*-acetylation masks sialoglycan ligands for Siglec-2, *Siglec2* knockout alone in mice was not sufficient to induce B cell abnormalities, but required additional knockout of *Siglecg,* probably due to redundant functions of these Siglecs (144). The role of sialic acid *O*-acetylation including SIAE, CASD1, and interactions with Siglecs in autoimmunity and inflammation is therefore open for investigation.

Next to masking effects and regulation of receptor interactions, sialic acid *O*-acetylation of glycolipids has been indicated to regulate immune cell development and activation. *O*-acetylated GD3 can be detected on human T cells, B cells, monocytes, and erythrocytes using antibodies specific for non-*O*-acetylated GD3 (CD60a), 7-*O*-Ac-GD3 (CD60c), or 9-*O*-Ac-GD3 (CD60b) (145, 146, 147, 148). Interestingly, anti-CD60b antibodies provide costimulatory signals to T cells and B cells and anti-CD60c antibodies induce lymphocyte proliferation (145,149, 150, 151). On the other hand, non-*O*-acetylated GD3 can be internalized by activated T cells and induces mitochondrial-mediated apoptosis (152). These studies suggest that *O*-acetylated GD3 and clustering thereof have anti-apoptotic effects and could contribute to immune cell development and activation, but this requires more detailed investigation. Recently, *Casd1* knockout mice were generated and showed loss of *O*-Ac-Sia in the immune cells; however, no abnormalities in hematopoiesis were found (85). Therefore, *O*-Ac-Sia may not be critical in immune development, but these mice now provide an opportunity for detailed dissection of the role of *O*-Ac-Sia in the immune response and infection.

## Microbiome and infection

### Sialoglycan degradation and microbiome interactions

Sialic acids can protect glycoproteins from glycosidase and protease activity, making desialylation an important first step in glycoprotein degradation. For example, desialylation of serum glycoproteins and platelets triggers clearance from circulation *via* the liver Ashwell–Morell receptor (11, 12, 13). There are four, and potentially more, mammalian sialidases designated NEU1-4 that cleave sialic acids from glycans in different cellular compartments and in circulation (153). *O*-acetylation potentially reduces activity of these sialidases (154) and may thereby regulate glycoprotein/glycolipid ageing and degradation. Hunter and coworkers developed a fluorogenic reporter assay to probe the effects of 9-*O*-acetylation on human NEU1-4 activity (155). NEU1 activity was too low to detect, but *O*-acetylation (Neu5,9Ac_2_) markedly reduced NEU2 and NEU3 activity and slightly enhanced NEU4 activity. This study was extended by measuring release of Neu5Ac and Neu5,9Ac_2_ from bovine submaxillary mucin, revealing a more than tenfold preference of NEU2-4 for Neu5Ac over Neu5,9Ac_2_ whereas NEU1 appeared more tolerant for *O*-Ac-Sias (156). Further studies should address the effects of *O*-acetylation on sialidase activity under consideration of the glycoconjugate context and the subcellular environment of NEU1-4. Notably, neuraminic acid pyruvate-lyase (NPL) that breaks down sialic acids into N-acetylmannosamine and pyruvate may be hindered by *O*-acetylation in a similar manner (6), warranting further research into the degradation and recycling of *O*-Ac-Sias.

**Figure 3:**
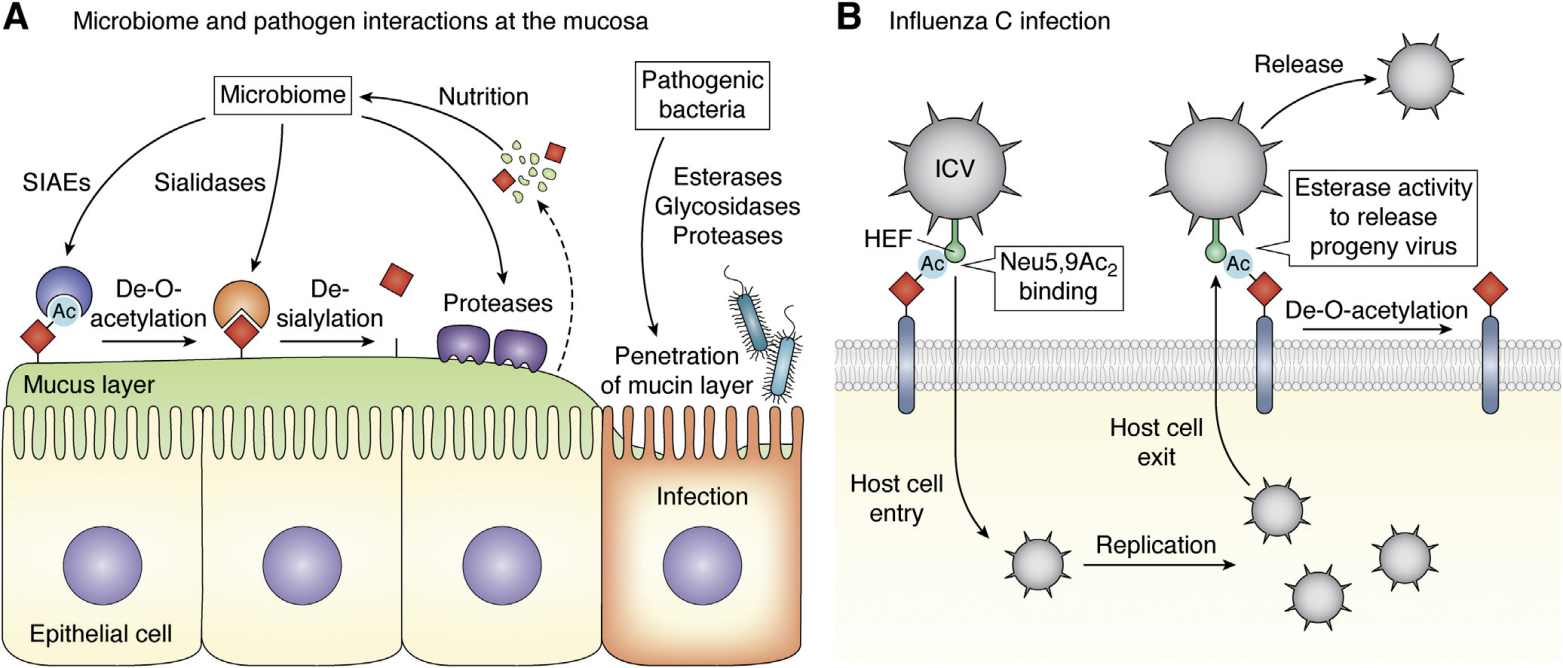
***O*-acetylated sialic acids in microbiome interactions and influenza C infection.***A*, the epithelial surfaces are covered with a mucus layer composed of densely O-glycosylated mucin glycoproteins that provide adhesion sites for the microbiome and are degraded for nutrition. Mucin O-glycans contain *O*-acetylated sialic acids that are resistant to bacterial sialidase activity. To achieve mucin degradation, SIAEs are produced by the microbiome that first deacetylate sialic acids followed by removal of sialic acids through sialidases. The mucins are now susceptible to the activity of other microbial glycosidases and proteases and released sugars and amino acids are used as nutrients by the microbiome. Mucin production by the epithelium and degradation through the microbiome are usually in balance; however, pathogens with high esterase, glycosidase, and protease activity can penetrate the mucus barrier for attachment to epithelial cells and infection. *B*, the influenza C virus (ICV) displays a hemagglutinin-esterase fusion (HEF) glycoprotein that mediates binding to Neu5,9Ac_2_-containing glycans at the host cell surface and cellular entry of the viral particle. After virus replication, the HEF esterase activity cleaves *O*-acetyl groups from surface sialic acids to facilitate release of progeny virus from the host cells.

Similar to its effects on mammalian sialidases, *O*-acetylation inhibits the activity of bacterial sialidases and serves in protecting the integrity of the epithelial mucus barrier (Fig. 3*A*). In the colon and at other epithelial surfaces, several bacteria can produce sialidases that initiate mucus breakdown by glycosidases and proteases, thereby generating nutrients and adhesion sites for the microbiome (157, 158, 159). The mucus layer in the intestinal tract is formed by gel-forming mucin 2 (MUC2), which is densely O-glycosylated, in humans mainly with core3 GalNAc-type O-glycans (GlcNAcβ1-3(NeuAcα2-6)GalNAc) with extension by Sd^a^/Cad epitopes (GalNAcβ1-4[NeuAcα2-3]Galβ1-R), sialylation, sulfation, and fucosylation (126, 160, 161). MUC2 is produced and secreted by specialized goblet cells and approximately half of the incorporated sialic acids are Neu5Ac, whereas the other half consists of Sia derivatives (73). Mass spectrometry and chromatography analysis of the sialic acid diversity of colonic mucin from healthy individuals determined that depending on the colonic region about 20% (ileum, cecum transverse, sigmoid) to 50% (rectum) of the total Sias are *O*-acetylated (73, 123, 125). This includes mono-, di-, and tri-*O*-acetylated Sias, and Neu5,9Ac_2_ was most frequently found. How *O*-acetylation of mucins is regulated in goblet cells is not clear, but interestingly these studies found comparable *O*-Ac-Sia levels between samples from different individuals (73, 123, 125). The group size in these studies was small (2–5 individuals), thus further investigation into the variability of *O*-Ac-Sia patterns of mucins and their regulation in goblet cells in the colon and other sites is required.

*O*-Ac-Sias such as Neu5,7Ac_2_ and Neu5,9Ac_2_ are partially and Neu4,5Ac_2_ is largely resistant to bacterial sialidases (162, 163). Several commensal bacteria produce SIAEs that deacetylate *O*-Ac-Sias, which renders them susceptible to microbial sialidases and subsequent degradation of the mucin layer (126, 162, 164, 165) (Fig. 3*A*). These studies suggest that sialic acid *O*-acetylation is an important regulator of the mucosa allowing balanced mucin degradation to sustain the microbiome while preserving barrier integrity. Notably, Kinoshita and colleagues recently suggested that aging and high-fat diet can change serum *O*-Ac-Sia levels (166), and it would be interesting to investigate how/if these factors alter mucosal *O*-Ac-Sia levels and mucus barrier integrity.

### Bacterial and parasite infection

In pathogenic bacteria, SIAE activity can contribute to virulence by facilitating mucin degradation and penetration of the mucus barrier resulting in infection of the epithelial cells (Fig. 3*A*, Table 1). For example, enterohemorrhagic *Escherichia coli* (EHEC) that can cause severe intestinal infection and hemorrhagic lesions produces SIAEs (NanS and NanS-p) capable of deacetylating *O*-Ac-Sias of colonic mucins. NanS has a broad substrate scope for Neu5Ac/Gc with mono-, di-, or tri-*O*-acetylation at C-7, C-8, and/or C-9, but not C-4 (167, 168, 169, 170, 171). For mucin degradation, EHEC secretes a potent mucinase, StcE (172, 173), and recently it was shown that the sialyl-Tn antigen (Neu5Acα2-6GalNAcα1-O-Ser/Thr) found on MUC2 protects against StcE activity (174). This suggests that mucin desialylation is required for this pathogen to penetrate the mucin barrier and although sialidase activity in *E. coli* has not been established so far, SIAE production by EHEC presumably facilitates the activity of other bacterial sialidases (165). *Streptococcus pneumoniae* has SIAE activity and additionally produces three sialidases (nanA/B/C), which can act synergistically in the degradation of the mucosal barrier in the respiratory tract to promote pneumoniae (175). Concerted action of SIAE and sialidase activity were also proposed in infection with *Tannerella forsythia* that colonizes the oral microbiome (176, 177). These studies suggest that deacetylation renders mucins susceptible to sialidases and subsequently other glycosidases and proteases from pathogens and commensals. The released glycans and amino acids serve as nutrients supporting pathogenic growth and the exposed intestinal epithelium is vulnerable to bacterial attachment and infection (Fig. 3*A*). This notion is further supported by recent studies reporting that enhanced microbial sialidase activity associates with intestinal inflammation in mice (178). Further research into how bacterial SIAE secretion and activity are regulated, their specificity for *O*-Ac-Sia forms, and interplay with other glycosidases from commensals and pathogens will provide rationale for application of SIAE inhibitors to support integrity of the mucosal barrier in infection. This research should also address how the pH at the mucosal surfaces influences migration of *O*-acetyl groups along the sialic acid glycerol chain and if this forms an additional resistance mechanism against pathogenic esterases and sialidases.

In typhoid fever, caused by the human pathogen Salmonella typhi, Neu5,9Ac_2_ is involved in the binding of typhoid toxin to host cells (179). *S. typhi* grows in the cytosol of host cells where it produces typhoid toxin (A_2_B_5_). The intracellular typhoid toxin can be transported to the extracellular environment where it binds and enters adjacent cells *via* the pentameric B_5_ subunit and the two enzymatically active A_2_ subunits induce DNA damage and cell cycle arrest (180). Glycan array studies revealed preference of the B_5_ subunit for trisaccharides on branched N-glycans with terminal α2-3-linked sialic acid (Neu5Acα2-3Galβ1-4GlcNAc) (181, 182) and especially for the 9-*O*-acetylated form, which makes additional interactions with the B_5_ subunit (179). Subsequently, HEK293 cells with *CASD1* knockout lacking *O*-acetylation were less susceptible to typhoid toxin compared to cells with *CASD1* overexpression (179). The extent to which other bacterial toxins and adhesins might have developed specificity for *O*-Ac-Sia and the glycoconjugate and glycoprotein context should be further addressed using for example (cell-based) glycan arrays (174, 183, 184, 185).

Several human pathogenic bacteria synthesize sialic acids (mainly Neu5Ac) or utilize host sialic acids for incorporation into their capsular polysaccharides (CPS) as molecular mimicry and immune resistance mechanism (18, 186, 187). Additionally, some species including *E. coli* K1, group B *streptococcus* (*Streptococcus agalactiae*), *Neisseria meningitidis*, and *Campylobacter jejuni* are able to install *O*-acetyl groups to C-7/8/9 by virtue of expressing SOATs and SIAEs. (Table 1) (188, 189, 190, 191, 192). For instance*, E. coli* K1 builds a linear polysialic acid capsule, the K1 antigen, formed by about 200 α2-8-linked sialic acids with 7/9-*O*-acetylation (188, 193). The SOAT neuO was identified to *O*-acetylate sialic acid polymers with a minimal chain length of 14 sialic acid residues using Ac-CoA as donor (194, 195, 196, 197). It is not clear if *O*-acetylation of the K1 polysialic acid chain proceeds in a processive or distributive fashion. Another SOAT (neuD) was proposed to acetylate monomeric sialic acids in *E. coli* K1, prior to glycoconjugate assembly, and a putative esterase (neuA) was suggested to regulate *O*-Ac-Sia levels (198, 199). The roles of (de-)acetylation are not fully understood, but may favor virulence of *E. coli* K1 possibly by increasing the hydrophobicity of the capsular sialic acids, which may reduce antigenicity, antimicrobial factor binding, and sialidase activity (200).

*N. meningitidis* is a human-specific neuroinvasive pathogen and the serogroups C, Y, and W135 produce *O*-acetylated capsular polysialic acids (Table 1). Capsules of the C serogroup contain α2-9-linked sialic acids that can be *O*-acetylated by the transferase OatC at the C-7/8 position (191, 201). Group Y and W135 capsular sialic acids are built from polymers with α2-6 sialic acid linked to glucose (Y) or galactose (W-135), respectively, and can be *O*-acetylated at C-7/9 by the transferase OatWY (192, 202). Similarly, *C. jejuni* that mediates food poisoning expresses an *O*-acetyltransferase (orf11) that acetylates capsular α2-8-linked sialic acids at C-9 (190). Presumably, *O*-acetylation changes the conformation and antigenicity of capsular polysialic acids that facilitate immune evasion in *N. meningitis* and *C. jejuni*, but this notion requires further experimental proof. In group B *streptococcus* (GBS), the functions of *O*-Ac-Sias are better understood. Nizet and coworkers detected α2-3-linked Neu5,7/8/9Ac_2_ on the outer capsular polysaccharides of various GBS serotypes and identified the underlying SOAT (neuD) as well as a SIAE (neuA) (189, 203, 204). The SOAT neuD acetylates monomeric sialic acids prior to glycoconjugate assembly (204). The bifunctional enzyme neuA is capable of producing CMP-sialic acids *via* its cytidyltransferase domain and can deacetylate monomeric *O*-Ac-Sia either prior to or after CMP activation using its esterase domain (203). Hence, the interplay of neuD and neuA regulates the levels of *O*-Ac-CMP-Sia building blocks that are used for production of the α2-3-linked Neu5,7/8/9Ac_2_ capping residues in GBS. The ability to vary the levels of *O*-Ac-Sia on its CPS may enable GBS to adapt to its environment. For example, GBS α2-3-linked Sias can frustrate antimicrobial activity of platelets and neutrophils *via* interactions with Siglec-9 (205, 206); however, *O*-acetylation seems to abolish these interactions (207, 208). Possibly, GBS uses dynamic (de-)*O*-acetylation to adapt to the different stages of infection. During intestinal and vaginal colonization, *O*-acetylation could protect the bacteria from sialidase or protease activity and immune recognition, and downregulation at the infection stage could expose the capsular sialic acids for Siglec interactions and suppression of the antimicrobial defense (209).

Notably, parasites also appear to utilize *O*-Ac-Sia to gain advantages during infection. Neu5,9Ac_2_ was identified on the cell surface of virulent forms of *Leishmania donovani* and may contribute to infection of macrophages, intercellular survival, and resistance against the host immune response (210, 211) (Table 1). Further understanding of bacteria and parasite utilization of *O*-Ac-Sia during infection is needed to develop strategies for therapeutic intervention and capsular *O*-Ac-Sias could become specific target molecules herein.

### Viral infection

The human airway epithelium contains 7/9-*O*-Ac-Sia-carrying glycans, which are utilized as binding sites, also referred to as receptors, by certain viruses for entry into host cells. This group includes the influenza and coronaviruses that will be briefly discussed here (Table 2). For more detailed information on the sialic acid–virus interplay, we refer to these recent reviews (19, 212, 213, 214, 215). Human Influenza A and B viruses (IAV, IBV) that cause the seasonal flu express hemagglutinin (HA) and sialidase (neuraminidase, NA) glycoproteins for binding to and release from non-*O*-acetylated sialic acids, respectively, with preference for α2-6-linked sialic acids displayed on N-glycans (3, 216, 217). In contrast to IAV and IBV, influenza C (ICV) and D (IDV) viruses display only one multifunctional hemagglutinin esterase fusion (HEF) glycoprotein on their surface, which mediates binding to Neu5,9Ac_2_ (and Neu5Gc9Ac), uptake into host cells, and deacetylation during release of progeny virus (88, 218, 219, 220, 221) (Fig. 3*B*). The X-ray crystal structure of ICV hemagglutinin shows that it contains a nonpolar pocket to accommodate the 9-*O*-Ac group and a relaxed linkage specificity as 9-*O*-Ac-Sias on α2-3-linked, α2-6-linked, and α2-8-linked sialosides are recognized, although with a strong preference for the latter (222, 223). The esterase domain in complex with a nonhydrolyzable 9-*O*-Ac-Sia analogue was also characterized *via* X-ray crystallography confirming the binding mode of the acetyl group in a nonpolar pocket (222). ICV infection causes mild respiratory symptoms in humans, and IDV that resides in cattle has not yet been detected in humans (224). The HEF glycoproteins are highly useful in biomedical research for removal of *O*-acetyl groups and enzymatically inactive forms serve as virolectins to probe *O*-Ac-Sia (87, 225).

**Table 2:** *O*-Ac-Sia utilization by human viruses

Virus family	Virus genus	*O*-Ac-Sia binding	SIAE/GBP	References
Orthomyxoviridae	Influenza C virus (ICV)	Neu5,9Ac_2_ (α2-8-linked, GL)	HEF	(218, 222, 223)
	Influenza D virus (IDV)	Neu5,9Ac_2_	HEF	(219, 221, 223)
Coronaviridae	Coronavirus OC43	Neu5,9Ac_2_ (α2-8-linked)	HE/S	(223, 230, 232)
	Coronavirus HKU1	Neu5,9Ac_2_ (α2-8-linked)	HE/S	(223, 227, 230, 231, 252)

Specificity for *O*-Ac-Sias and viral surface proteins involved in binding and hydrolysis of *O*-Ac-Sias are listed.

Abbreviations: GL, glycolipids; HE, hemagglutinin esterase; HEF, hemagglutinin-esterase fusion protein; S, virus spike protein.

Similar to influenza viruses, coronaviruses can cause mild to severe respiratory infections, and outbreaks occurred several times upon transmission from animals to humans. Coronaviruses use a trimeric spike glycoprotein on the viral envelope to bind and fuse with host cells, and some family members recognize sialic acids (215). The 2012 Middle East respiratory syndrome coronavirus (MERS-CoV) recognizes α2-3-linked Neu5Ac as major ligand and 7/9-*O*-acetylation blocks binding (226). On the other hand, both endemic human coronaviruses HCoV-OC43 and HCoV-HKU1 infect the upper respiratory tract and can cause the common cold bind Neu5,9Ac_2_, preferably on α2-8-sialosides (223, 227, 228, 229, 230) (Table 2). Like all coronaviruses, HCoV-OC43 and HCoV-HKU1 display a long (20 nm) trimeric spike glycoprotein (S), and additionally they express a shorter (8 nm) hemagglutinin esterase (HE) glycoprotein (230). The S protein mediates binding to host cell 9-*O*-Ac-Sias and membrane fusion for viral entry, whereas the HE protein deacetylates *O*-Ac-Sias during release of viral progeny from the host cell surface and possibly also for breaking decoy interactions of the S protein with *O*-Ac-Sia carrying mucins (227, 228). Notably, HE proteins from other viruses such as ICV contain an active lectin domain that mediates glycan binding, but the HE lectin domains of HCoV-OC43 and HCoV-HKU1 lost glycan-binding ability due to mutations and deletions during adaptation to the human host (227, 228), although their esterase activity was maintained (229). Recently, it was shown that the diminished lectin activity of HCoV-OC43 and HCoV-HKU1 HE is taken over by the lectin domain in the S protein that mediates low-affinity binding to 9-*O*-Ac-Sia with a binding mode similar to that of ICV HEF (230, 231, 232). Presumably, this lectin domain “switch” is an adaptation to the human sialome.

The 2002/2003 severe acute respiratory syndrome coronavirus (SARS-CoV-1) and the 2019/2020 SARS-CoV-2 bind ACE2 as protein ligand (233, 234). Heparin sulfate proteoglycans have been identified as codeterminant for SARS-CoV-2 infection (235), and the role of glycans should be further addressed. Interactions of the SARS-CoV-2 spike protein with sialic acids have been suggested (236), but it remains questionable if they are of relevance for host cell infection and involvement of *O*-Ac-Sia is unlikely since both SARS-CoV strains lack the hemagglutinin esterase or related domains (237). Regarding the reservoir of influenza and coronaviruses residing in animal hosts that have *O*-acetyl sialic acid–binding capacity (238), further research into virus *O*-Ac-Sia interactions is important for prevention of zoonotic transmission.

## Chemical synthesis and inhibitor development

### Chemical tools to probe sialic acid *O*-acetylation

Dissecting the relationship of the sialic acid acetylation pattern and its biological relevance is challenging due to the number of mono-, di-, and tri-*O*-acetyl forms of sialic acid that can exist. This is compounded by additional complexity arising from sialic acid linkage types (α2-3, α2-6, and α2-8) and the structure of the underlying glycoconjugate. Furthermore, due to the chemical lability of the acetyl esters, the synthesis of reference compounds is challenging. This challenge was recently overcome by the (chemoenzymatic) synthesis of a panel of well-defined mono-*O*-acetylated sialic acids (239), *O*-acetylated CPS (240), and mammalian host-derived glycoconjugates (223) containing *O*-Ac-Sias. For example, human or animal-derived sialoglycans with α2-3, α2-6, or α2-8-linked mono-, di-, and tri-*O*-acetylated Sias were prepared from stable tri-*O*-Ac-Sia precursors using selective enzymatic deacetylation by HEs of differing specificity and pH-controlled chemical acetyl migration (223). The resulting panel of well-defined *O*-acetylated sialoglycans was printed on a microarray and screened against inactivated viral HE(F)s and S proteins originating from various mammalian viruses. Quality control experiments confirmed that spontaneous acetyl migration did not occur during microarray printing or storage. HE(F)s and S proteins from pathogens that infect the human host (OC43, HKU1, and ICV) showed a strong preference for α2-8-linked 9-*O*-Ac-Sias (223). Subsequent binding experiments with human cell lines and tissues identified α2-8-linked 9-*O*-Ac-Sias in gangliosides as shared binding site/entry receptor for these pathogens. This microarray can further enable dissection of the preferred *O*-Ac-Sia ligands for other human and animal viruses and may help to identify other glycan-binding proteins with specificity for *O*-Ac-Sias.

**Figure 4:**
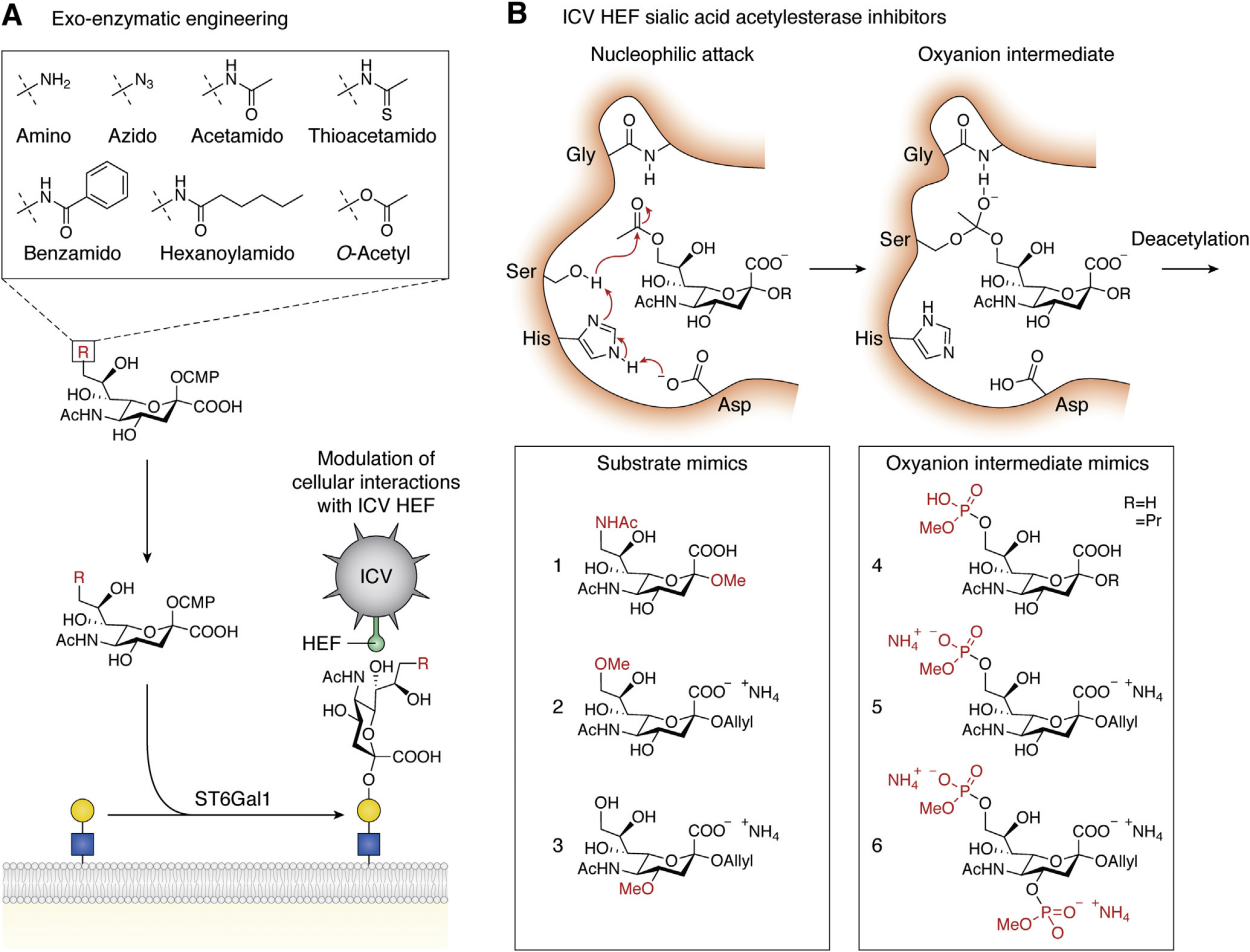
**Exoenzymatic installation of *O*-acetylated sialic acids to cell surface glycans and overview of SIAE inhibitors**. *A*, unnatural C-9-modified CMP-sialic acids or *O*-acetylated CMP-sialic acids (*box*) can be enzymatically conjugated to free galactose residues (*yellow circles*) at the cell surface using recombinant ST6Gal1 sialyltransferase. Modifications with the unnatural C-9 derivatives can alter recognition of the influenza C virus (ICV) hemagglutinin esterase fusion (HEF) protein and its esterase activity. *B*, *upper panel* shows the catalytic site of the ICV HEF and the mechanism of de-*O*-acetylation of Neu5,9Ac_2_ starting with a nucleophilic attack of the hydroxyl group (OH) from the serine (Ser) on the sialic acid C-9 carbonyl group (*left*), which is assisted by the aspartic acid (Asp) and histidine (His) that form the catalytic triad. The oxyanion intermediate is stabilized in the catalytic center including interactions with the glycine (Gly) residue (*right*). The *lower panel* shows structures of reported substrate mimics (compounds 1–3) and oxyanion intermediate mimics (compounds 4–6).

A different exoenzymatic engineering approach to investigate the structural determinants for ICV HEF binding and esterase activity utilized unnatural sialic acid analogues (Fig. 4*A*). Synthetic CMP-sialic acid analogues varying in the substituents at position C-9 were synthesized with amino, azido, acetamido, thioacetamido, benzamido, hexanoylamido, and acetyl groups, respectively (241, 242, 243, 244). Using recombinant ST6GAL1, these derivatives were transferred with comparable efficiencies (except for the C-9 amino derivative) to the surface of chicken red blood cells or canine MDCK cells to assess the interactions with ICV HEF (Fig. 4*A*). ICV can bind Neu5,9Ac_2_ *via* its HEF protein resulting in agglutination of red blood cells. Modification of red blood cells with the unnatural 9-acetamido and 9-thioacetamido derivatives maintained ICV-mediated agglutination, but required 5–10-fold higher cell surface concentrations compared with Neu5,9Ac_2_ indicating that these derivatives are less efficiently recognized by the HEF protein (241, 242, 243, 244). All other tested C-9 modifications were not recognized by the HEF protein, rendering red blood cells resistant to agglutination by ICV (241, 242, 243, 244). In line with these findings, preincubation of red blood cells modified with 9-*O*-Ac or 9-acetamido Sias with purified HEF acetyl esterase rendered the 9-*O*-Ac-modified cells resistant to agglutination, but not the 9-acetamido-modified cells (241). This shows that the 9-acetamido sialic acids are resistant to the HEF esterase activity, which was also validated with HPLC analysis of purified sialic acids (241). Interestingly, modification of erythrocytes with 9-*O*-Ac-Sias or C-9 derivatives had no effect on ICV fusion and subsequent erythrocyte lysis, suggesting that 9-*O*-Ac-Sias are involved in ICV binding to host cells, but not fusion (241, 242). Contrarily, while 9-*O*-Ac-Sia-modified MDCK cells were efficiently infected with ICV, modification with the 9-thioacetamido derivative blocked infection indicating that the HEF esterase activity is required for infection (242). It is furthermore likely that the esterase activity of HEF is important for the escape of viral progeny from the host cells, because chemoenzymatic transfer of 9-thioacetamido Sias onto influenza C virions led to aggregation due to virion–virion interactions that were resistant to esterase activity and led to a >10-fold reduction in infectivity (244). Compared with glycan arrays, chemoenzymatic modification of living cells with 9-*O*-Ac-Sias and derivatives lacks structural insight into specific glycoconjugates involved in binding, but this approach enables functional biological assays that can advance understanding of the role of *O*-Ac-Sias in viral infection and other processes.

In a more recent study, 9-*O*-acetyl and 9-N-acetamido-modified Sias were incorporated as metabolic precursors in live human cells omitting the need for exoenzymatic incorporation (36). A human lymphoma cell line was cultured with 1–3 mM concentrations of the sialic acid analogue and both showed incorporation as assayed with an inactivated porcine torovirus esterase. The 9-acetamido derivative showed a better incorporation and stability over time compared with the 9-*O*-acetyl derivative, which is presumably partially deacetylated inside cells (36). This metabolic oligosaccharide engineering strategy represents a simple method to probe the role of sialic acid C-9 acetylation and may be extended to other positions of the sialic acid core.

### Inhibitors of the sialic acid acetyl esterase

The growing number of studies linking *O*-Ac Sias to disease strongly suggest that inhibition of SOATs or SIAEs has a broad therapeutic potential. Inhibitors of CASD1 might be useful to target sialic acid *O*-acetylation in cancer to facilitate GD3-mediated apoptosis, to trigger immune inhibitory Siglec signaling in autoimmunity, or to increase the immunogenicity of capsular sialic acids in bacteria. SIAE inhibitors hold the promise to reinforce the epithelial mucus barrier during infection and inflammation and may reduce coronavirus or influenza C/D virus infection. So far, only a few strategies for inhibition of SIAEs are available and inhibitors for SOATs have not been reported yet. Early work by Palese *et al.* demonstrated the use of fluorophosphate, benzoxazinones, isocoumarins, boronic acids, and trifluoromethyl ketones as inhibitors of the bovine coronavirus (BCoV) and ICV esterases (245, 246). These inhibitors are broad-spectrum covalent inhibitors that generally interact with the active site serine of serine hydrolases and are hence not selective for SIAE. Brossmer *et al.* reported on the synthesis of a C-9 *N*-acetyl sialic acid α-methyl glycoside (Fig. 4*B*, compound 1) and its properties as an inhibitor of the ICV HEF esterase. This molecule mimics the substrate of ICV HEF, 9-*O*-Ac-Sia, but proved to be a very weak inhibitor with an inhibitory constant of ∼2.8 mM (241, 247, 248). In a subsequent study, Wong *et al.* attempted to develop more potent inhibitors by mimicking the oxyanion intermediate state formed after nucleophilic attack of the catalytic serine (247). To this end, a methylphosphodiester was installed at the C-9 position of sialic acid (Fig. 4*B*, compound 4). The tetrahedral and anonic group was expected to be a good transition state mimic fitting the “oxyanonic hole” within the enzyme; however, the observed inhibition constant was only ∼2 mM. To investigate the influence of the aglycone on the inhibitory potency, an α-propyl sialoside derivative was prepared, but showed little improvement (249). More recently, Streicher *et al.* reported a follow-up study evaluating the inhibitory potency of sialosides modified with a methyl phosphodiester (Fig. 4*B*, compounds 5, 6) or methyl ether (Fig. 4*B*, compounds 2, 3) positioned at C-9 or C-4 for inhibition of esterase activity of ICV, BCoV, and murine hepatitis virus strain S (MHV-S) HE as well as two recombinant chimeric HEs from ICV (HE-12) and sialodacryoadenitis virus (SDAV-HE) (250). The methyl phosphodiester derivatives (Fig. 4*B*, compounds, 5, 6) showed little to no inhibition of HE activity regardless of the position. From the methyl ether derivatives, only the C-9 derivative (Fig. 4*B*, compound 2) showed minor inhibition of the recombinant HE12 esterase (85% inhibition at 5 mM), and no inhibitory activity on the viral HEs was observed. Although these first-generation SIAE inhibitors lack potency and selectivity, refinement of the underlying approach of using sialosides with chemical modifications of the glycerol chain may allow selective targeting of bacterial and viral SIAEs. Further development and screening of sialoside libraries may identify lead compounds that potently interfere with SIAE activity and further structure-guided optimization could yield high-affinity inhibitors for SIAE targeting in bacterial and viral infection. Such libraries could be readily developed over the next few years based, for example, on the recent *O*-Ac-Sia microarray library (223), and structures for several bacterial and viral SIAEs are already available (169, 222, 228, 251, 252, 253) that should guide the design of the next generation of inhibitors. A similar strategy should be applied for the development of the first SOAT inhibitors.

## Conclusion and prospective

Single or multiple *O*-acetyl modifications at the C-4/7/8/9 position of sialic acids are frequently found in nature and contribute to the diverse functions and interactions of sialoglycans. Although many aspects of *O*-Ac-Sia biosynthesis and biology remain to be explored, research over the past decades has paved the way for recent technical advances in the study of *O*-Ac-Sias that will place these versatile molecules more into the focus of biological and medical research. In particular the identification of CASD1 and SIAE has greatly advanced the understanding of mammalian *O*-Ac-Sia biosynthesis and now drives the generation of genetically engineered cell lines and model organisms with gene knockout/in or chemical modifications that will enable discovering the molecular and cellular functions of these enzymes and *O*-Ac-Sias. Functional assays, based on genetically or chemoenzymatically engineered cells, recombinant enzymes, or synthetic libraries of *O*-acetylated sialoglycans are emerging that allow dissecting the effects of *O*-acetylation on sialoglycan-active enzymes and glycan-binding proteins interacting with sialoglycans such as sialyltransferases, sialidases, or Siglecs, respectively. Likewise, advances in the detection, analysis, and synthesis of the different *O*-Ac-Sia forms now allow comprehensive investigations of their species, organ, and cell-type-specific expression and specific interactions, for example, with influenza C/D or coronaviruses. Furthermore, the implications of *O*-Ac-Sias in cancer and infection may spark the development of the first SOAT inhibitors and the second generation of selective and potent inhibitors for targeting of mammalian or bacterial SIAEs and viral hemagglutinin esterase that could prove highly useful in a therapeutic setting.

## Conflict of interest

The authors declare that they have no conflicts of interest with the contents of this article.
